# Effect of induction agent on vasopressor and steroid use, and outcome in patients with septic shock

**DOI:** 10.1186/cc5916

**Published:** 2007-05-16

**Authors:** David Charles Ray, Dermot William McKeown

**Affiliations:** 1 Department of Anaesthesia, Critical Care & Pain Medicine, Royal Infirmary of Edinburgh, Little France Crescent, Edinburgh EH16 4SA, Scotland, UK

## Abstract

**Introduction:**

In seriously ill patients, etomidate gives cardiovascular stability at induction of anaesthesia, but there is concern over possible adrenal suppression. Etomidate could reduce steroid synthesis and increase the need for vasopressor and steroid therapy. The outcome could be worse than in patients given other induction agents.

**Methods:**

We reviewed 159 septic shock patients admitted to our intensive care unit (ICU) over a 40-month period to study the association between induction agent and clinical outcome, including vasopressor, inotrope, and steroid therapy. From our records, we retrieved induction agent use; vasopressor administration at induction; vasopressor, inotrope, and steroid administration in the ICU; and hospital outcome.

**Results:**

Hospital mortality was 65%. The numbers of patients given an induction agent were 74, etomidate; 25, propofol; 26, thiopental; 18, other agent; and 16, no agent. Vasopressor, inotrope, or steroid administration and outcome were not related to the induction agent chosen. Corticosteroid therapy given to patients who received etomidate did not affect outcome. Vasopressor therapy was required less frequently and in smaller doses when etomidate was used to induce anaesthesia. We found no evidence that either clinical outcome or therapy was affected when etomidate was used. Etomidate caused less cardiovascular depression than other induction agents in patients with septic shock.

**Conclusion:**

Etomidate use for critically ill patients should consider all of these issues and not simply the possibility of adrenal suppression, which may not be important when steroid supplements are used.

## Introduction

In patients with sepsis, induction of anaesthesia can be hazardous. Hypoxaemia, hypotension, volume depletion, and renal impairment may be present. No currently available induction agent is ideal. Possible agents are propofol, thiopental, etomidate, midazolam, and ketamine. In non-septic patients, cardiovascular depression is greatest with propofol [1,2], but thiopental can also cause significant hypotension [1-3]. Etomidate causes less cardiovascular depression than propofol or thiopental [1,2], but it can suppress adrenal function through blockade of 11β-hydroxylase [4-7]. This suppression persists for at least 24 hours [8,9], and some authors suggest that it may last up to 72 hours [10]. This could harm patients with critical illness such as severe sepsis or septic shock.

Etomidate has been scrutinised with regard to its safety in critically ill patients [11-16]. Much of this debate has been fuelled by opinion rather than clear evidence of deleterious clinical effect. Etomidate undoubtedly causes adrenal suppression, but the clinical consequences of this are not clear. Adrenal suppression in critical illness is controversial, particularly 'relative' adrenal insufficiency [17-19]. The incidence of adrenal suppression in septic shock ranges from 9% to 67% [18,20-22], but there is little evidence that adrenal suppression is related to outcome [8,20,23,24]. Cortisol response to corticotrophin is more frequently impaired in critically ill patients given etomidate [8,9], including those with septic shock [23], than those who receive an alternative induction agent. Retrospective analyses suggest that etomidate may be associated with increased mortality in septic patients [10,25]. Corticosteroid treatment of these patients appeared to improve outcome [10], although steroid was administered in a randomised fashion rather than specifically to treat hypotension that did not respond to vasopressors. Annane [10] found circumstantial evidence for a clinically deleterious effect of etomidate on adrenal function; septic patients given etomidate received more fluid and vasopressor therapy than those given other induction agents [10,26]. If adrenal suppression were clinically important in the critically ill, patients given etomidate would require more vasopressor and steroid support and would have worse outcome than patients who received an alternative induction agent.

Most comment has been on the adverse effects of etomidate. However, this agent also has potential benefits. A formal randomised study would allow full evaluation but would be difficult to perform. We have a substantial database that can provide an indication of the value of such a study. To study the association between induction agent and (a) the use of vasopressor, inotrope, and steroids and (b) outcome, we retrospectively analysed the data from septic shock patients admitted over several years to a large general intensive care unit (ICU).

## Materials and methods

### Setting

The chairman of the local research and ethics committee stated that formal approval and informed consent were not required for this retrospective review. We studied patients admitted to an 18-bed adult ICU in a major teaching and tertiary referral centre. The unit admits patients with critical illness except those after cardiac surgery, those with uncomplicated cardiological problems, and those with isolated head injury. The unit admits 1,036 patients per year (averaged over the past three years), and 715 (69%) require intensive care (level 3) rather than high-dependency care (level 2). The average APACHE II (Acute Physiology and Chronic Health Evaluation II) scores are 18.4 for all admissions and 20.4 for level 3 patients. Six hundred fourteen (59%) patients receive ventilatory support, and 159 (15%) require renal replacement therapy. We use the Scottish Intensive Care Society WardWatcher™ database to record details such as reason for admission, diagnosis and patient outcome, and predicted outcome.

Steroid treatment is used for septic shock patients who respond poorly to vasopressor agents. We use a protocol that requires that hydrocortisone 100 mg be given every eight hours if a patient with sepsis remains hypotensive (mean arterial pressure of less than 65 mm Hg or systolic blood pressure of less than 90 mm Hg) despite vasopressor or if the dose of noradrenaline exceeds 0.28 μg/kg per minute. We do not routinely measure plasma cortisol concentration or perform corticotrophin stimulation tests. All patients in this review were managed according to this protocol.

### Patients

We reviewed all patients admitted between 1 April 2003 and 31 August 2006. During this period, we admitted 3,554 patients, and 2,054 of these required level 3 care. WardWatcher™ identified 242 patients with a diagnosis of septic shock, and 208 of these required tracheal intubation and ventilation. We obtained the case notes for 192 of these patients; case notes were not available for the remaining 16 patients. We excluded 33 patients from analysis. In 13, we could not identify the induction agent that had been used, and 10 had been intubated in another hospital before transfer to our unit; an additional 10 patients were recorded as having septic shock but required no vasopressor therapy. Complete information was therefore available for 159 patients. Patient characteristics are shown in Table 1.

**Table 1:** Characteristics of 159 patients for whom complete information was available

Male/female	90:69
Age in years (standard deviation)	65 (14)
APACHE II score (range)	27 (11–53)
Predicted mortality (range)	67% (11%–99%)
Intensive care unit mortality	60%
Hospital mortality	65%
Intensive care unit length of stay in days (range)	5.2 (0.1–65)
Percentage given steroids	55%
Source of sepsis, number (percentage)	
Pulmonary	51 (32%)
Gastrointestinal	63 (40%)
Renal	5 (3%)
Unspecified	40 (25%)
Medical/surgical patients	86:73

Data are given as numbers, mean (standard deviation), or median (range). APACHE II, Acute Physiology and Chronic Health Evaluation II.

### Review design

We recorded patient details, source of sepsis, admission and outcome details, diagnoses, the highest SOFA (Sequential Organ Failure Assessment) score (minus the neurological component) in the first seven days of ICU admission, induction agent given, dose and duration of vasopressor or inotropic support, and dose of steroid administered. We noted whether the patient was receiving an infusion of vasopressor or inotrope at the time of induction of anaesthesia and whether any significant cardiovascular problems had been documented at induction.

### Statistical analysis

We used one-way analysis of variance, the Mann-Whitney *U *test, and the Kruskal-Wallis test as appropriate to assess differences between patients given different induction agents. Analysis of differences in outcome and therapy between groups was performed using the χ^2 ^test. We considered a *P *value of less than 0.05 to be statistically significant. We used Minitab commercial software (version 12.1; Minitab Inc., State College, PA, USA).

## Results

Complete data were available for analysis in 159 patients. The agents (number of patients) used to induce anaesthesia were etomidate (74), propofol (25), thiopental (26), midazolam (14), ketamine (1), and fentanyl (1). Two patients had inhalational induction of anaesthesia with sevoflurane because of coexisting acute upper airway obstruction. Sixteen patients received no agent to induce anaesthesia; 14 of these had tracheal intubation during cardiopulmonary resuscitation for cardiac arrest, and two patients had awake fibreoptic intubation. We combined the data for patients given midazolam, ketamine, fentanyl, or inhalational induction into a group entitled 'other'. The median doses (range) of agents administered were etomidate, 12 (5 to 20) mg; propofol, 60 (20 to 180) mg; thiopental, 200 (75 to 450) mg; and midazolam, 2 (2 to 3) mg. Eighty-four patients were intubated in the ICU and 75 were intubated in areas outside the ICU, mostly in an operating theatre or the emergency department. One hundred forty-nine (94%) patients were intubated within six hours of ICU admission and eight others were intubated within 24 hours of admission. All 159 patients were intubated because of septic shock; 153 (96%) were intubated within 24 hours of the onset of shock, an additional five were intubated within 48 hours, and in one patient tracheal intubation occurred 78 hours after the onset of sepsis.

**Figure 1:**
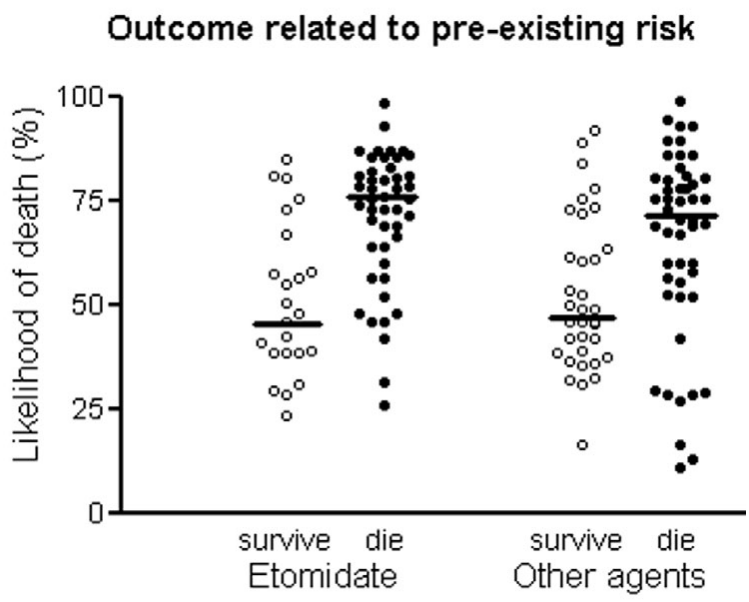
Outcome related to Acute Physiology and Chronic Health Evaluation II predicted mortality for patients given etomidate and those given other agents. Horizontal bar represents the median value.

Severity of illness and outcome are shown in Table 2. Patients given thiopental appeared to be less severely ill and have better survival than patients in any other group, but these differences did not reach statistical significance. Outcome related to pre-existing risk was similar for patients given etomidate and those given other agents (Figure 1).

**Table 2:** Details of severity of illness and outcome for each induction agent

	Etomidate (*n *= 74)	Propofol (*n *= 25)	Thiopental (*n *= 26)	Other (*n *= 18)	Nil (*n *= 16)	*P *value
Mean age in years	65	63	66	66	66	0.35
APACHE II score	28	24	24	29	30	0.70
Predicted mortality	69%	57%	52%	71%	75%	0.49
Hospital mortality	69%	56%	46%	67%	81%	0.23
SOFA score	10	10	8	11	10	0.40
Crude SMR	1.0	0.98	0.88	0.94	1.08	

Except for age, data shown are median values. APACHE II, Acute Physiology and Chronic Health Evaluation II; SMR, standardised mortality ratio; SOFA, Sequential Organ Failure Assessment.

All 159 patients received vasoactive infusions. These were noradrenaline (*n *= 153), dobutamine (*n *= 52), adrenaline (*n *= 39), and vasopressin (*n *= 3). The mean numbers of vasoactive infusions per patient were 1.6, etomidate; 1.5, propofol; 1.4, thiopental; 1.6, other; and 1.8, no agent. Choice of induction agent was not related to timing of commencing noradrenaline; duration of noradrenaline infusion; total, maximum, or averaged noradrenaline dose (Table 3); or averaged dobutamine dose (data not shown).

**Table 3:** Details of noradrenaline therapy received by patients in each group

	Etomidate	Propofol	Thiopental	Other	Nil	*P *value
Number (percentage) receiving	72 (97%)	25 (100%)	26 (100%)	17 (94%)	14 (88%)	
Total dose in milligrams	46	44	52	84	65	0.53
Maximum dose in micrograms per kilogram per minute	0.45	0.40	0.38	0.50	0.46	0.64
Averaged dose in micrograms per kilogram per minute	0.26	0.23	0.19	0.30	0.32	0.29
Duration of infusion in hours	52	59	47	50	40	0.54
Time from intubation to commencing infusion in minutes	68	135	105	15	230	0.49

Data shown are median values.

Eighty-seven patients received hydrocortisone for vasopressor-dependent hypotension. No patient had plasma cortisol concentration measured or corticotrophin tests performed. Twelve other patients had been taking prednisolone for chronic respiratory or musculoskeletal problems or following organ transplantation. Nine were given intravenous hydrocortisone, and three continued prednisolone. Of the 87 patients who started steroid therapy, 58 (67%) died; of the 60 patients who received no steroid, 36 (60%) died. Patients who received hydrocortisone tended to be more severely ill and were more likely to have medical rather than surgical pathology (Table 4). The median time from induction of anaesthesia to first hydrocortisone dose for all 87 patients was 11 hours, and the median time from commencing noradrenaline to first steroid dose was 9 hours. The induction agent used did not influence subsequent steroid administration, dose of hydrocortisone, or timing of administration (Table 5). Forty-three patients given etomidate received steroids; 32 (74%) died compared with 19 (58%) who died and did not receive steroid (*P *= 0.121).

**Table 4:** Characteristics of patients given hydrocortisone and those who received no steroid

	Hydrocortisone (*n *= 87)	No steroid (*n *= 60)	*P *value
Male/female	54:33	32:28	0.29
Mean age in years	66	65	0.90
APACHE II score	28	26	0.49
Predicted mortality	70%	60%	0.42
SOFA score	11	9	0.014
Medical/surgical	49:38	29:31	0.34
Total NA dose in milligrams	73	31	< 0.001
Maximum NA dose in micrograms per kilogram per minute	0.57	0.26	< 0.001
Averaged NA dose in micrograms per kilogram per minute	0.31	0.15	< 0.001

Data for the 12 patients taking prednisolone chronically are not included. Except for age, data are given as numbers or median. NA, noradrenaline; SOFA, Sequential Organ Failure Assessment.

**Table 5:** Details of hydrocortisone therapy received by patients in each group

	Etomidate	Propofol	Thiopental	Other	Nil	*P *value
Number (percentage) receiving	39 (53%)	14 (56%)	17 (65%)	12 (67%)	9 (56%)	0.74
Total dose in milligrams	600	700	500	600	400	0.46
Duration of therapy in hours	60	64	40	48	32	0.45
Time from intubation to first dose in hours	10	9	17	4	5	0.36

Data shown are median values.

**Figure 2:**
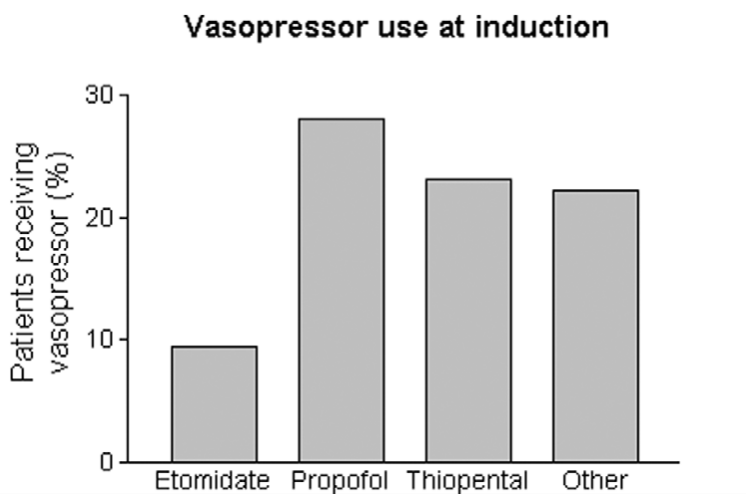
Percentage of patients given bolus dose of vasopressor at induction of anaesthesia, grouped by induction agent.

Of the 143 patients given an induction agent, 26 were receiving an infusion of a vasoactive agent (usually noradrenaline) at the time of induction. Thirteen of these patients received etomidate, four received propofol, one received thiopental, and six received an 'other' agent. In the 143 patients who received an induction agent, 23 required bolus administration of vasoactive agents during induction of anaesthesia. After etomidate administration, vasopressor use appeared to be less frequent, but this was not significant (Figure 2), and there was less active management of cardiovascular depression during induction of anaesthesia compared with propofol or other agent (Table 6).

**Table 6:** Intensity of cardiovascular management at induction of anaesthesia

	Minor	Moderate	Intensive
Etomidate	●●●●	●●●	
Propofol	●	●	●●●●●
Thiopental	●●●	●	
Midazolam			●●●●

Each dot represents one patient. Minor: less than or equal to 1 mg of metaraminol or less than or equal to 6 mg of ephedrine; moderate: more than 1 mg to less than or equal to 3 mg of metaraminol or more than 6 mg to less than or equal to 18 mg of ephedrine; intensive: more than 3 mg of metaraminol or any dose of adrenaline.

## Discussion

We found that induction agent did not affect subsequent therapy with vasopressor, inotrope, and steroid, and outcome. Patients given etomidate and steroid had greater mortality than those who received etomidate alone. This contrasts with reports that patients given etomidate received more subsequent vasopressor support than patients given other induction agents and that administration of steroid to those who received etomidate improved outcome [10]. Our indication for steroid treatment was lack of sustained response to vasopressor and not lack of response to corticotrophin stimulation testing. Interpretation of stimulation tests is very difficult in critical illness and does not accurately and consistently identify patients who might benefit from steroid administration. Choice of steroid and duration of therapy may be important. We gave only hydrocortisone, whereas a previous study by Annane and colleagues [26] used hydrocortisone and fludrocortisone; the benefit of additional fludrocortisone is not known. It has been suggested that steroid therapy should be continued for 5 to 11 days to have full effect [27]. The median time from ICU admission to death in our patients who received steroid was only 1.8 days compared with 19.5 days in the other study [26]. Perhaps our patients did not survive long enough to gain full benefit from steroid administration, but our patients had similar survival to those in the study [26] in which steroids improved survival. The form of vasopressor therapy also differed between the studies; more of our patients received noradrenaline (90.5% versus 30.3%), and the median duration of therapy was much shorter (51 hours versus 7 to 9 days). Such differences may reflect the different rationales for commencing steroids in the two studies. We cannot confirm that steroid treatment improves outcome in septic patients given etomidate.

Hospital mortality for the 159 patients in the study was 65%, which is comparable with rates found in other studies of septic shock [28-30]. Patients given etomidate were sicker than those given propofol or thiopental and were less likely to survive. When the standardised mortality ratios (SMRs) (actual hospital mortality/predicted APACHE II mortality) are calculated for each of these groups, outcome is not significantly affected by the induction agent. Thus, etomidate did not have a demonstrable adverse effect on outcome. However, the SMR was higher for etomidate (1.0) than in the other pooled groups (0.96). Although this difference is relatively small, it is possible that etomidate may be associated with a worse 'adjusted' outcome. Despite concerns about etomidate-induced adrenal suppression, etomidate was chosen more frequently for sicker patients, and this did not lead to increased use of vasopressors, inotropes, or steroids. The dose of etomidate given in the present study (approximately 0.1 to 0.3 mg/kg) is lower than that given in other studies [9,15,26]. It is possible that the amplitude of adrenal suppression is dose-related [16] and that we might have observed a greater difference if we had used larger doses of etomidate. However, even a sub-anaesthetic dose of 0.04 mg/kg can block 11 β-hydroxylase [31], and we are not aware of any evidence that the clinical consequences of adrenal suppression following a single bolus of etomidate are dose-related. Patients who received hydrocortisone to treat vasopressor-dependent hypotension appeared to be sicker than patients who received no steroid therapy. This may account for our finding that outcome was worse in these patients, but it could be argued that steroids should improve patients more substantially if the vasopressor-dependence is related mainly to adrenal suppression.

Tracheal intubation in critically ill patients can cause immediate and severe life-threatening complications [32,33]. Patients with hypotension are particularly at risk [32-34] and are more likely to die after tracheal intubation than are normotensive patients [34]. Hypotension at induction is a common feature in anaesthesia-related deaths [35]. Etomidate may be especially useful in critically ill and hypotensive patients because it has little effect on systemic blood pressure [1-3,36-39]. After etomidate, fewer patients required vasopressor agents at induction and less cardiovascular intervention was required than in patients given propofol, thiopental, or other agents. Etomidate appears to cause less cardiovascular depression than propofol or thiopental in critically ill septic patients.

We recognise the limitations of retrospective reviews. A prospective study with randomisation of induction agent might address some of these limitations, but such a study may be difficult to undertake. WardWatcher™ is an excellent, nationally co-ordinated and audited ICU database, which provides the best possible method of obtaining data and assessment, short of undertaking a prospective study. In our review, some patients may have been misdiagnosed with septic shock and others may have been missed if the diagnosis of septic shock was not entered into the WardWatcher™ database. However, we believe ascertainment bias was small. We identified 208 septic shock patients who required tracheal intubation. This accounts for 10% of the patients admitted for intensive care during the review period, giving a prevalence of septic shock similar to other studies [27,40]. Case notes were not available for 16 patients, and an additional 23 patients were excluded from analysis because of missing data or because induction of anaesthesia had occurred in another hospital. Twenty-six of these patients (67%) died and 36 received vasoactive therapy (92%). It is therefore unlikely that data from the missing patients would significantly alter the main findings of our review.

## Conclusion

We conclude that induction agent use cannot be related to patient outcome, vasoactive use, or steroid use in this particular cohort of patients. Steroid treatment for vasopressor-dependent hypotension in patients who received etomidate did not improve survival. There are cogent reasons for choosing etomidate for induction in patients with impaired cardiovascular status. Bolus vasoactive therapy is required less frequently at induction with etomidate; if such therapy is required, the doses used are lower than after other agents. The use of etomidate in critically ill patients should consider all of these issues rather than the single aspect of adrenal suppression.

## Key messages

In this review of septic shock patients admitted to our intensive care unit:

• Vasopressor use and dose are not influenced by induction agent.

• Steroid use for vasopressor-resistant hypotension is not influenced by induction agent.

• Outcome related to pre-existing risk is not different if etomidate is used for induction.

• Outcome is not improved if steroids are administered to patients given etomidate.

• Induction with etomidate causes less cardiovascular depression than other induction agents.

## Abbreviations

APACHE II = Acute Physiology and Chronic Health Evaluation II; ICU = intensive care unit; SMR = standardised mortality ratio.

## Competing interests

The authors declare that they have no competing interests.

## Authors' contributions

DCR designed the study; acquired, analysed, and interpreted the data; and drafted the manuscript. DWM conceived of the study, participated in its design and coordination, and helped to draft the manuscript. Both authors read and approved the final manuscript.

## References

[B1] McCollum JSC, Dundee JW (1986). Comparison of induction characteristics of four intravenous anaesthetic agents. Anaesthesia.

[B2] Benson M, Junger A, Fuchs C, Quinzio L, Bottger S, Hempelmann G (2000). Use of an anesthesia information management system (AIMS) to evaluate the physiologic effects of hypnotic agents used to induce anesthesia. J Clin Monit Comput.

[B3] Reich DL, Hossain SMA, Krol M, Baez B, Patel P, Bernstein A, Bodian CA (2005). Predictors of hypotension after induction of general anesthesia. Anesth Analg.

[B4] Duthie DJR, Fraser R, Nimmo WS (1985). Effect of induction of anaesthesia with etomidate on corticosteroid synthesis in man. Br J Anaesth.

[B5] Wagner RL, White PF, Kan PB, Rosenthal MH, Feldman D (1984). Inhibition of adrenal steroidogenesis by the anesthetic etomidate. N Engl J Med.

[B6] Watt I, Ledingham IM (1984). Mortality amongst multiple trauma patients admitted to an intensive therapy unit. Anaesthesia.

[B7] Ledingham I, Watt I (1983). Influence of sedation on mortality in critically ill multiple trauma patients. Lancet.

[B8] Absalom A, Pledger D, Kong A (1999). Adrenocortical function in critically ill patients 24 h after a single dose of etomidate. Anaesthesia.

[B9] Malerba G, Romano-Girard F, Cravoisy A, Dousset B, Nace L, Levy B, Bollaert P-E (2005). Risk factors of relative adrenocortical deficiency in intensive care patients needing mechanical ventilation. Intensive Care Med.

[B10] Annane D (2005). Etomidate and intensive care physicians. Intensive Care Med.

[B11] Annane D (2005). ICU physicians should abandon the use of etomidate!. Intensive Care Med.

[B12] Morris C, McAllister C (2005). Etomidate for emergency anaesthesia: mad, bad and dangerous to know?. Anaesthesia.

[B13] Bloomfield R, Noble DW (2006). Etomidate and fatal outcome – even a single bolus dose may be detrimental for some patients. Br J Anaesth.

[B14] Bloomfield R, Noble DW (2006). Etomidate, pharmacological adrenalectomy and the critically ill: a matter of vital importance. Crit Care.

[B15] Jackson WL (2005). Should we use etomidate as an induction agent for endotracheal intubation in patients with septic shock?: a critical appraisal. Chest.

[B16] Murray H, Marik PE (2005). Etomidate for endotracheal intubation in sepsis: acknowledging the good while accepting the bad. Chest.

[B17] Keh D (2006). Corticosteroid therapy in sepsis: where are we?. Adv Sepsis.

[B18] Marik PE, Zaloga GP (2003). Adrenal insufficiency during septic shock. Crit Care Med.

[B19] Cooper MS, Stewart PM (2003). Current concepts: corticosteroid insufficiency in acutely ill patients. N Engl J Med.

[B20] Pizarro C, Troster EJ, Damiani D, Carcillo JA (2005). Absolute and relative adrenal insufficiency in children with septic shock. Crit Care Med.

[B21] Annane D, Sebille V, Troche G, Raphael JC, Gajdos P, Bellissant E (2000). A 3-level prognostic classification in septic shock based on cortisol levels and cortisol response to corticotropin. JAMA.

[B22] Siraux V, De Backer D, Yalavatti G, Melot C, Gervy C, Mockel J, Vincent J-L (2005). Relative adrenal insufficiency in patients with septic shock: comparison of low-dose and conventional corticotrophin tests. Crit Care Med.

[B23] Mohammad Z, Afessa B, Finkielman JD (2006). The incidence of relative adrenal insufficiency in patients with septic shock after the administration of etomidate. Crit Care.

[B24] Young SP, Newman L Response to 'Morris C, McAllister C: Etomidate for emergency anaesthesia: mad, bad and dangerous to know? *Anaesthesia *2005, 60:737–740.'. Anaesthesia.

[B25] Den Brinker M, Joosten KF, Liem O, de Jong FH, Hop WC, Hazelzet JA, van Dijk M, Hokken-Koelega AC (2005). Adrenal insufficiency in meningococcal sepsis: bioavailable cortisol levels and impact of interleukin-6 levels and intubation with etomidate on adrenal function and mortality. J Clin Endocrinol Metab.

[B26] Annane D, Sebille V, Charpentier C, Bollaert P-E, Francois B, Korach J-M, Capellier G, Cohen Y, Azoulay E, Troche G (2002). Effect of treatment with low doses of hydrocortisone and fludrocortisone on mortality in patients with septic shock. JAMA.

[B27] Annane D, Bellissant E, Bollaer't PE, Briegel J, Keh D, Kupfer Y (2004). Corticosteroids for severe sepsis and septic shock: a systematic review and meta-analysis. BMJ.

[B28] Dellinger RP (2003). Cardiovascular management of septic shock. Crit Care Med.

[B29] Friedman G, Silva E, Vincent JL (1998). Has the mortality of septic shock changed with time?. Crit Care Med.

[B30] Angus D, Linde-Zwirble WT, Lidicker J, Clermont G, Carcillo J, Pinsky MR (2001). Epidemiology of severe sepsis in the United States: analysis of incidence, outcome, and associated costs of care. Crit Care Med.

[B31] Diago MC, Amado JA, Otero M, Lopez-Cordovilla JJ (1988). Anti-adrenal action of a subanaesthetic dose of etomidate. Anaesthesia.

[B32] Jaber S, Amraoui J, Lefrant J-Y, Arich C, Cohendy R, Landreau L, Calvet Y, Capdevila X, Mahamat A, Eledjam J-J (2006). Clinical practice and risk factors for immediate complications of endotracheal intubation in the intensive care unit: a prospective, multiple-center study. Crit Care Med.

[B33] Leibowitz AB (2006). Tracheal intubation in the intensive care unit: extremely hazardous even in the best of hands. Crit Care Med.

[B34] Schwartz DE, Matthay MA, Cohen NH (1995). Death and other complications of emergency airway management in critically ill adults: a prospective investigation of 297 tracheal intubations. Anesthesiology.

[B35] Arbous MS, Grobbee DE, van Kleef JW, de Lange JJ, Spoormans HHAJM, Touw P, Werner FM, Meursing AEE (2001). Mortality associated with anaesthesia: a qualitative analysis to identify risk factors. Anaesthesia.

[B36] Reynolds SF, Heffner J (2005). Airway management of the critically ill patient: rapid-sequence intubation. Chest.

[B37] Bergen JM, Smith DC (1997). A review of etomidate for rapid sequence intubation in the emergency department. J Emerg Med.

[B38] Oglesby AJ (2004). Should etomidate be the induction agent of choice for rapid sequence intubation in the emergency department?. Emerg Med J.

[B39] Sprung J, Ogletree-Hughes ML, Moravec CS (2000). The effects of etomidate on the contractility of the failing and nonfailing human heart muscle. Anesth Analg.

[B40] Annane D, Aegerter P, Jars-Guincestre MC, Guidet B (2003). Current epidemiology of septic shock: the CUB-Réa Network. Am J Respir Crit Care Med.

